# Poly(benzoquinonyl sulfide) as a High‐Energy Organic Cathode for Rechargeable Li and Na Batteries

**DOI:** 10.1002/advs.201500124

**Published:** 2015-06-08

**Authors:** Zhiping Song, Yumin Qian, Tao Zhang, Minoru Otani, Haoshen Zhou

**Affiliations:** ^1^ Energy Technology Research Institute (ETRI)National Institute of Advanced Industrial Science and Technology (AIST)305‐8568TsukubaJapan; ^2^ Nanosystem Research Institute (NRI)National Institute of Advanced Industrial Science and Technology (AIST)305‐8568TsukubaJapan; ^3^ Elements Strategy Initiative for Catalysts and Batteries (ESICB)Kyoto University615‐8520KyotoJapan; ^4^ National Laboratory of Solid State MicrostructuresDepartment of Energy Science and EngineeringNanjing University210093NanjingChina

**Keywords:** lithium batteries, organic cathodes, polymers, quinones, sodium batteries

## Abstract

In concern of resource sustainability and environmental friendliness, organic electrode materials for rechargeable batteries have attracted increasing attentions in recent years. However, for many researchers, the primary impression on organic cathode materials is the poor cycling stability and low energy density, mainly due to the unfavorable dissolution and low redox potential, respectively. Herein, a novel polymer cathode material, namely poly(benzoquinonyl sulfide) (PBQS) is reported, for either rechargeable Li or Na battery. Remarkably, PBQS shows a high energy density of 734 W h kg^–1^ (2.67 V × 275 mA h g^–1^) in Li battery, or 557 W h kg^–1^ (2.08 V × 268 mA h g^–1^) in Na battery, which exceeds those of most inorganic Li or Na intercalation cathodes. Moreover, PBQS also demonstrates excellent long‐term cycling stability (1000 cycles, 86%) and superior rate capability (5000 mA g^–1^, 72%) in Li battery. Besides the exciting battery performance, investigations on the structure–property relationship between benzoquinone (BQ) and PBQS, and electrochemical behavior difference between Li–PBQS battery and Na–PBQS battery, also provide significant insights into developing better Li‐organic and Na‐organic batteries beyond conventional Li‐ion batteries.

## 1 Introduction

Battery technology is now recognized as a main bottleneck of the development of consumer electronics, electric vehicles, and energy storage stations.^1^, ^2^ The situation is that conventional Li‐ion batteries meet the ceiling of energy density because of the limited choices of inorganic intercalation cathodes,^3^ while Li–S and Li–O_2_ batteries still remain many fundamental problems to address, in spite of their attractive energy densities.^4^, ^5^, ^6^ As alternatives, Li‐organic batteries are full of potential to achieve not only significant advantages in resource sustainability and environmental friendliness,^1^, ^7^, ^8^, ^9^ but also excellent electrochemical performance including high energy density. Especially, the recent intensive investigations on conjugated‐carbonyl‐based organic cathode materials (quinones,^10^, ^11^, ^12^, ^13^, ^14^, ^15^, ^16^, ^17^, ^18^, ^19^, ^20^, ^21^ dianhydrides,^22^, ^23^, ^24^ carboxylates,^25^, ^26^, ^27^, ^28^ diketones,^29^, ^30^ etc.), have made huge progress to demonstrate their enormous possibilities in high energy density, long cycle life, and excellent high rate capability.

As we know, the energy density of a cathode material is determined by the average discharge voltage and the discharge specific capacity. For a conjugated‐carbonyl‐based organic cathode material, the redox potential is usually between 1.5 and 3.0 V versus Li^+^/Li, while the theoretical specific capacity is up to 600 mA h g^−1^,^8^, ^9^ so it is possible to gain a practical energy density up to above 1000 W h kg^−1^ [approximate to that of *x*Li_2_MnO_3_·(1−*x*)LiMO_2_, which is the highest for inorganic cathodes], if ignoring the unfavorable dissolution of active material in the electrolyte,^10^ or inhibiting it by using solid electrolytes.^15^, ^19^, ^20^ However, for more practical application in common liquid electrolyte with good long‐term cycling stability, it is necessary to develop organic cathodes with intrinsic insolubility or slight solubility, including organic polymers or organic salts. Due to the feasibility of synthesis, such kind of materials demonstrate energy densities restricted at the level of commercial inorganic cathodes (LiCoO_2_ and LiFePO_4_, 500–600 W h kg^−1^), for example, PPYT^29^ (2.5 V, 230 mA h g^−1^), Li_4_C_8_H_2_O_6_ 27 (2.6 V, 220 mA h g^−1^) and Li_2_PDHBQS^21^ (2.0 V, 270 mA h g^−1^).

The structure diversity of organic materials encourages us to develop organic cathode materials with both higher energy density and better cycling stability. Our strategy is to design and synthesize insoluble quinone‐based polymers possessing both high redox potential and high theoretical capacity. Benzoquinone (BQ) unit is one of the best choices on building blocks because of its superiority in both aspects^13^, ^15^, ^19^, ^20^ (≈2.7 V × 496 mA h g^−1^ = ≈1339 W h kg^−1^) and good availability for practical use. However, due to the high reactivity of benzoquinone monomers, the polymerization is rather difficult and no chemical synthesis of such polymers with high benzoquinone density was reported as far as we know. Herein, based on our understanding on the polycondensation reaction between halogenated quinone and sulfide,^12^, ^21^ and various attempts on the reaction conditions, we developed a polymerization–oxidation approach to synthesize a novel benzoquinone polymer, namely poly(benzoquinonyl sulfide) (PBQS), containing abundant benzoquinone units linked by thioether bonds. Compared to our previously studied Li_2_PDHBQS^21^ with similar structure, the absence of enolate groups adjacent to quinone groups can not only largely improve the theoretical capacity from 295 to 388 mA h g^−1^, but also greatly elevate the redox potential by about 0.6 V. As a result, PBQS shows a remarkable improvement in energy density (2.67 V × 275 mA h g^−1^ = 734 W h kg^−1^) of Li‐organic battery. Due to the specialty of conjugated‐carbonyl and the elevated redox potential, PBQS can be also directly applied as cathode for Na‐organic battery,^24^, ^26^, ^28^ gaining higher energy density (2.08 V × 268 mA h g^−1^ = 557 W h kg^−1^) than those of most inorganic cathodes for Na‐ion batteries.^31^

## 2 Results and Discussion

### 2.1 Synthesis and Characterization of PBQS

**Scheme 1:**
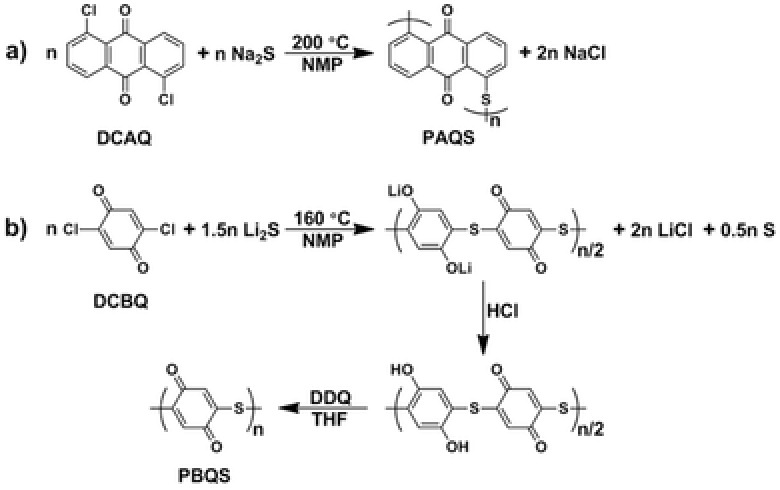
a) The previous synthesis route of PAQS. b) Proposed synthesis route of PBQS including the polycondensation reaction between DCBQ and Li_2_S, acidification by HCl, and oxidation by DDQ.

The structure of PBQS (**Scheme** **1**b) was proposed as one of the ideal structures of quinone‐based polymer cathodes, when we successfully synthesized poly(anthraquinonyl sulfide) (PAQS, Scheme 1a) and investigated it as cathode material for rechargeable Li battery in 2009.^12^ However, it is not as easy as expected to synthesize it and simple transplantation of the synthesis procedure to halogenated benzoquinone monomer failed to obtain a polymer product. The reason is that besides the polycondensation reaction between halogenated quinone monomers and sulfide anions, there also exists a side redox reaction between quinone groups and sulfide anions, depending on their relative reduction potential. Since benzoquinone group is much more inclined to oxidize sulfide anion than anthraquinone group,^9^ the polycondensation reaction will be seriously disturbed and result in low polymerization degree and troublesome product. After carefully optimizing the reaction conditions including the stoichiometric ratio of sulfide (Li_2_S) to monomer (dichloro​benzoquinone, DCBQ), solvent and temperature, we proposed a new polymerization reaction between DCBQ and Li_2_S as shown in Scheme 1b. Compared to the reaction between dichloro​anthraquinone (DCAQ) and Na_2_S to synthesize PAQS (Scheme 1a), the main difference is that 0.5*n* excess sulfide was used to reduce benzoquinone group, producing a polymer product in semireduction state. The electron‐donation effect of generated enolate anions will weaken the oxidizing ability of remaining benzoquinone groups (this will be discussed later in detail), preventing them from further reduction by sulfide anions. Due to the relatively low polymerization degree, the viscous mixture is troublesome to treat directly, which was acidified by HCl to separate the product, since such kind of phenolate tends to precipitate in acid solution.^21^ The precipitate was further purified by a salification–protonation approach (see the Experimental Details, Supporting Information), obtaining a black solid with high yield of ≈90%. In order to oxidize as‐prepared sample for our target material, we chose 2,3‐dichloro‐5,6‐dicyano‐1,4‐benzoquinone (DDQ) as oxidant since it is well‐known in the dehydrogenation of phenols.^32^ This oxidation step is possible to convert the majority of the hydroquinone groups into quinone groups, with yield close to 100%.

Various characterization methods were applied to confirm the structure of PBQS. First, elemental analysis was conducted for a preliminary estimation. The measured C, H, O, S, N, and Cl contents in PBQS after oxidation are respectively, 47.42%, 1.23%, 22.60%, 23.0%, 0.62%, and 0.91%, indicating that the C/S, C/O, and C/H molar ratios are respectively, 5.5, 2.8, and 3.2, which are roughly consistent with the theoretical values of 6, 3, and 3 (Scheme 1b). The Cl content is significantly reduced from 40.06% in monomer DCBQ to only 0.91% in PBQS, meaning that most of the Cl atoms were substituted by S atoms to form a polymer chain. Calculated from the C/S and C/Cl molar ratios (5.5 and 154), we can deduce that the average polymerization degree of PBQS is about 8, and the terminal groups are SH in the majority (≈84%) and Cl in the minority (≈16%). The detected N element (0.62%) clues the existence of residual 1‐methyl‐2‐pyrrolidone (NMP) solvent (≈4%) in the final product, which is difficult to be completely removed in the synthesis of polymers.^21^

**Figure 1:**
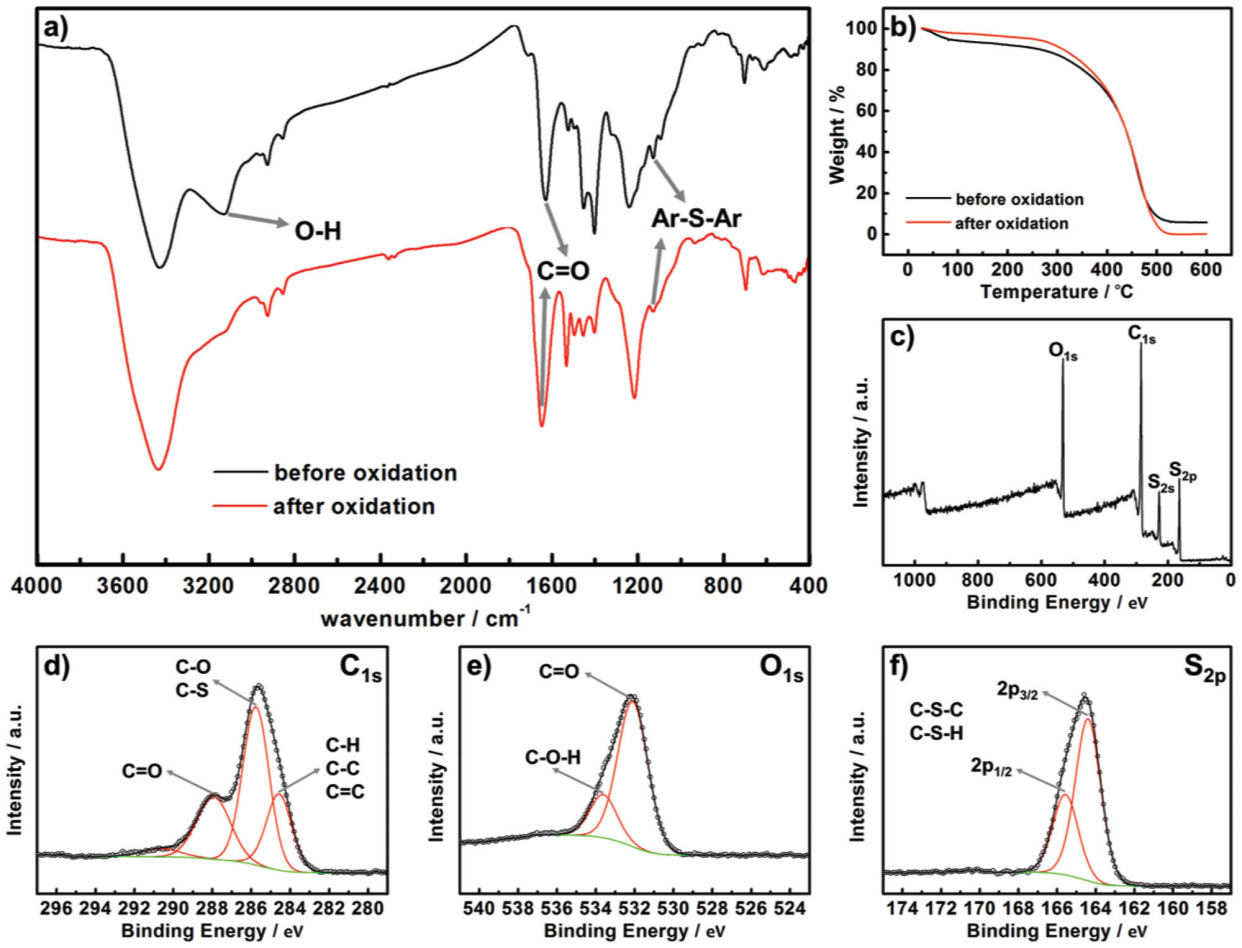
a) FTIR spectra of PBQS samples before and after oxidation. b) TG curves of PBQS samples before and after oxidation in air atmosphere at a heating rate of 5 °C min^–1^. XPS spectra of PBQS sample after oxidation: c) full range; d) C_1*s*_; e) O_1*s*_; f) S_2*p*_.

FTIR (Fourier‐transform infrared) spectra of PBQS samples were recorded to compare the structures before and after oxidation (**Figure** **1**a). The main characteristic peaks are well assigned in Table S1 (Supporting Information). Generally, from the similarity of their spectra, it is concluded that the polymer skeleton is well retained after oxidation. The weak absorption peak at 1127 or 1128 cm^–1^, and the multipeaks between 1400 and 1540 cm^–1^, which can be assigned to the stretching vibration of Ar–S bond^12^, ^21^, ^33^, ^34^ and sulfur‐substituted benzene ring,^12^, ^33^ respectively, together prove the formation of thioether bonds as linkers in the skeleton. The strong absorption peaks of C=O (at 1629 or 1647 cm^–1^, probably overlapping the peak of C=C^21^) and C–OH (at 1239 or 1216 cm^–1^), are observed for both PBQS samples, indicating that hydroquinone units still exist even after oxidation. However, the disappearance of the broad peak of O–H (at 3129 cm^–1^) before oxidation, combined with the increase of relative intensity of C=O (at 1647 cm^–1^) after oxidation, verifies that the majority of hydroquinone units were converted into quinone units after oxidation by DDQ. Due to the existence of unoxidized hydroquinone, absorbed water is also inevitable, as deduced from the broad and strong peak at 3427 or 3434 cm^–1^.

It is observed that the weight of PBQS is spontaneously increased after vacuum drying by absorbing moisture in air atmosphere, which is more obvious before oxidation than after oxidation. This is also a common property of other phenolates,^10^, ^21^ which can be ascribed to the tendency of enolate to form hydrogen bond (O–H···O) or coordination bond (O···Li···O) with H_2_O. Thermogravimetric (TG) analysis was performed to estimate the content of absorbed water in PBQS (Figure 1b). Assuming that the weight loss within 200 °C is mostly due to the evaporation of absorbed water, the content of absorbed water in stable PBQS phase stored in air atmosphere is 8% before oxidation or 4% after oxidation, respectively. The obvious decrease of H_2_O content can be also regarded as an indirect evidence of the conversion of hydroquinone into quinone.

XPS (X‐ray photoelectron spectroscopy) analysis was also conducted for PBQS sample after oxidation (Figure 1c–f). Generally, C, O, and S were detected as major elements, while the signals of N and Cl were negligible (Figure 1c), which is agreed with the above elemental analysis. For C_1*s*_ spectrum (Figure 1d), the three peaks at binding energy of 284.6, 285.8, and 288.0 eV can be assigned to unsubstituted C in benzene ring, C–S (O) and C=O, respectively. For O_1*s*_ spectrum (Figure 1e), the peak can be divided into two peaks at 532.1 eV for quinone (C=O) and 533.6 eV for hydroquinone (C–O–H), with calculated atom ratios of ≈78% and ≈22%, respectively. For S_2*p*_ spectrum (Figure 1f), the divided two peaks with gap of 1.2 eV (2*p*_3/2_ at 164.4 eV and 2*p*_1/2_ at 165.6 eV) both refer to C–S–C(H) bond. The above XPS analysis agree well with the FTIR spectra, and based on all the characterization results, we can conclude that our final PBQS product is a polymer containing benzoquinone units in the majority and hydroquinone units in the minority linked by thioether bonds, with unavoidable residual NMP and absorbed H_2_O in it. Although this is not a perfect structure as we proposed in Scheme 1b, it is still a significant progress if considering that no chemical synthesis of such kind of benzoquinone‐based polymers was reported so far.

### 2.2 PBQS Versus BQ

**Figure 2:**
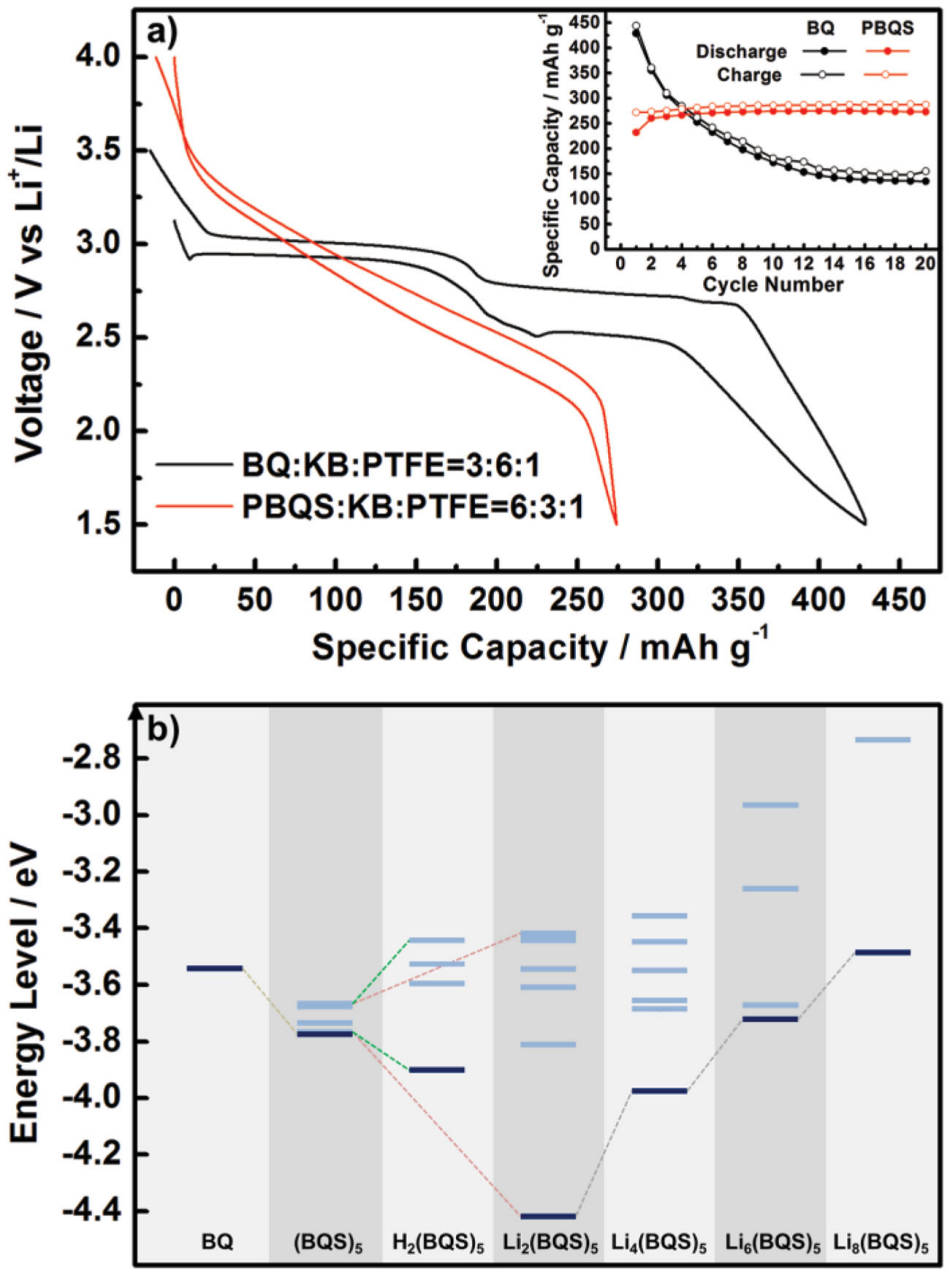
a) Typical discharge/charge curves of BQ (1.5–3.5 V, 1st cycle) and PBQS (1.5–4.0 V, 10th cycle) under the same current rate of 50 mA g^–1^. Inserted graph shows corresponding cycling performance within 20 cycles. Note that much lower BQ loading (30%) was used to restrain its dissolution in the electrolyte. b) Energy level diagram obtained by DFT calculation for the monomer (BQ), simulated polymer containing five structure units [(BQS)_5_], simulated polymer with residual hydroquinone group [H_2_(BQS)_5_], and in different reduction states [Li_2_(BQS)_5_, Li_4_(BQS)_5_, Li_6_(BQS)_5_, Li_8_(BQS)_5_]. Dark blue lines are the LUMOs and light blue lines are characteristic nearby unoccupied orbitals.

BQ is one of the most attractive organic cathode materials due to its high redox potential (2.5–3.0 V vs Li^+^/Li) and high theoretical capacity (496 mA h g^−1^). However, its serious volatility and solubility in organic solvents make it impossible to be directly applied in conventional Li battery with liquid electrolyte.^13^, ^15^, ^17^ **Figure** **2**a shows the typical battery performance of BQ cathode in 1 m LiTFSI/DOL + DME [LiN(CF_3_SO_2_)_2_ solution in a mixture of 1,3‐dioxolane and dimethoxyethane with volume ratio of 1:1] electrolyte. Even with a lowered active material loading of only 30%, the cycling stability of BQ is still very poor that the capacity retention is below 32% after 20 cycles, in spite of the very high initial discharge capacity (429 mA h g^−1^). As expected, after polymerization, PBQS cathode with 60% loading exhibits dramatically improved cycling stability, with high reversible capacity of 275 mA h g^−1^. This result once again proves that polymerization through thioether bonds is a simple but effective approach to guarantee the cycling stability while not to reduce the capacity too much.^12^, ^21^

In Figure 2a, it is observed that BQ shows two discharge/charge plateaus at 2.9 and 2.5 V, respectively, while PBQS presents just a sloping curve within a wide range of 3.4–2.1 V, leading to a slightly higher average discharge voltage (2.67 V) than that of BQ (2.54 V). It is interesting that PBQS starts to be reduced (at 3.4 V) much earlier than BQ (at 2.9 V), and the whole reduction process lasts for so large a voltage range (1.3 V). The different electrochemical behaviors of BQ and PBQS can be explained qualitatively from the structure differences of monomer and polymer, combined with electron inductive effect of neighboring groups. For BQ monomer or its derivatives, the two discharge/charge plateaus with small voltage gap,^13^, ^15^ declare its well‐known two‐step reaction mechanism involving BQ/BQ^•–^ and BQ^•–^/BQ^2–^ couples. For PBQS, the electron‐withdrawing effect of neighboring C=O groups can elevate the oxidizing ability of a specified BQ unit in the polymer chain, leading to a promoted starting reduction potential. But during the discharge process, more and more produced enolate groups (C–O^•–^) will gradually lower the oxidizing ability of remaining BQ units by electron‐donating effect, resulting in a sloping discharge curve.

The different electrochemical behaviors were also studied semiquantitatively by theoretical calculation. Since lowest unoccupied molecular orbital (LUMO) energy level is an efficient parameter to estimate the relative redox potential of an organic electrode material^13^, ^16^, ^18^, ^21^, ^29^, ^30^ (lower LUMO energy level indicates higher oxidizing ability and thus higher reduction potential), we have calculated the electron configurations of related molecules by DFT (density functional theory) method (Figure 2b, molecular formulas, geometries, and more detailed electron configurations can be found in Figures S1–S3, Supporting Information). For simplicity, we first used a short polymer chain containing *n* structure units, namely (BQS)*_n_* (*n* = 3, 5, 7, and 9), to simulate the structure of PBQS (Figure S1, Supporting Information). Compared to the LUMO energy of BQ monomer (–3.54 eV), (BQS)*_n_* shows *n* lowered and nearly degenerate orbitals (energy level width = 0.09–0.12 eV), with descending LUMO energies of –3.74, –3.77, –3.79, and –3.80 eV for (BQS)_3_, (BQS)_5_, (BQS)_7_, and (BQS)_9_, respectively (Figure S2, Supporting Information), suggesting that higher polymerization degree will slightly promote the starting reduction potential of PBQS. Considering both the representativeness of polymer structure and the complexity of calculation, we chose (BQS)_5_ as simulated PBQS for our further calculation. We were also concerned about polymers with residual hydroquinone groups [H_2_(BQS)_5_], and in different reduction states [Li_2_(BQS)_5_, Li_4_(BQS)_5_, Li_6_(BQS)_5_, and Li_8_(BQS)_5_], in the structures of which O–H and O···Li bonds were symmetrically and homogeneously distributed in the chain (Figure S1, Supporting Information). When we check the effect of residual hydroquinone units, it is surprising that the LUMO energy is lowered from –3.77 eV of (BQS)_5_ to –3.90 eV of H_2_(BQS)_5_ (Figure 2b), contrary to our thought that O–H will elevate the LUMO energy, as a reduction product of C=O. Similarly, Li_2_(BQS)_5_ shows even much lower LUMO energy of –4.42 eV. This abnormal phenomenon for H_2_(BQS)_5_ and Li_2_(BQS)_5_ is probably due to the pseudo‐Jahn−Teller effect (PJTE),^35^ which leads to the further splitting of pseudodegenerate LUMO energy levels of (BQS)_5_ (Figure 2b) to have more energy gain by molecular geometry distortion (Figure S1, Supporting Information), when electrons are injected. During the discharge process, as more and more Li ions are inserted into the cathode, LUMO energy of the molecule is gradually shifted from –4.42 eV of Li_2_(BQS)_5_ to –3.98 eV of Li_4_(BQS)_5_, –3.72 eV of Li_6_(BQS)_5_, and –3.49 eV of Li_8_(BQS)_5_. The negative LUMO energy shift from BQ to H_2_(BQS)_5_ (–0.36 eV), and positive LUMO energy shift from Li_2_(BQS)_5_ to Li_8_(BQS)_5_ (0.93 eV), can roughly agree with the starting reduction potential rise from BQ to PBQS (0.5 V), and the wide discharge potential range (1.3 V) of PBQS, respectively. Thus the different discharge behaviors of BQ and PBQS can be well illustrated by the simplified model we built, considering only the molecular geometric structure and ignoring many other factors such as intermolecular interaction, crystal structure, electrolyte, etc. We believe this calculation method is a simple but effective approach to predict the electrochemical behavior of such kind of polymer electrodes.

### 2.3 Li–PBQS Battery

**Figure 3:**
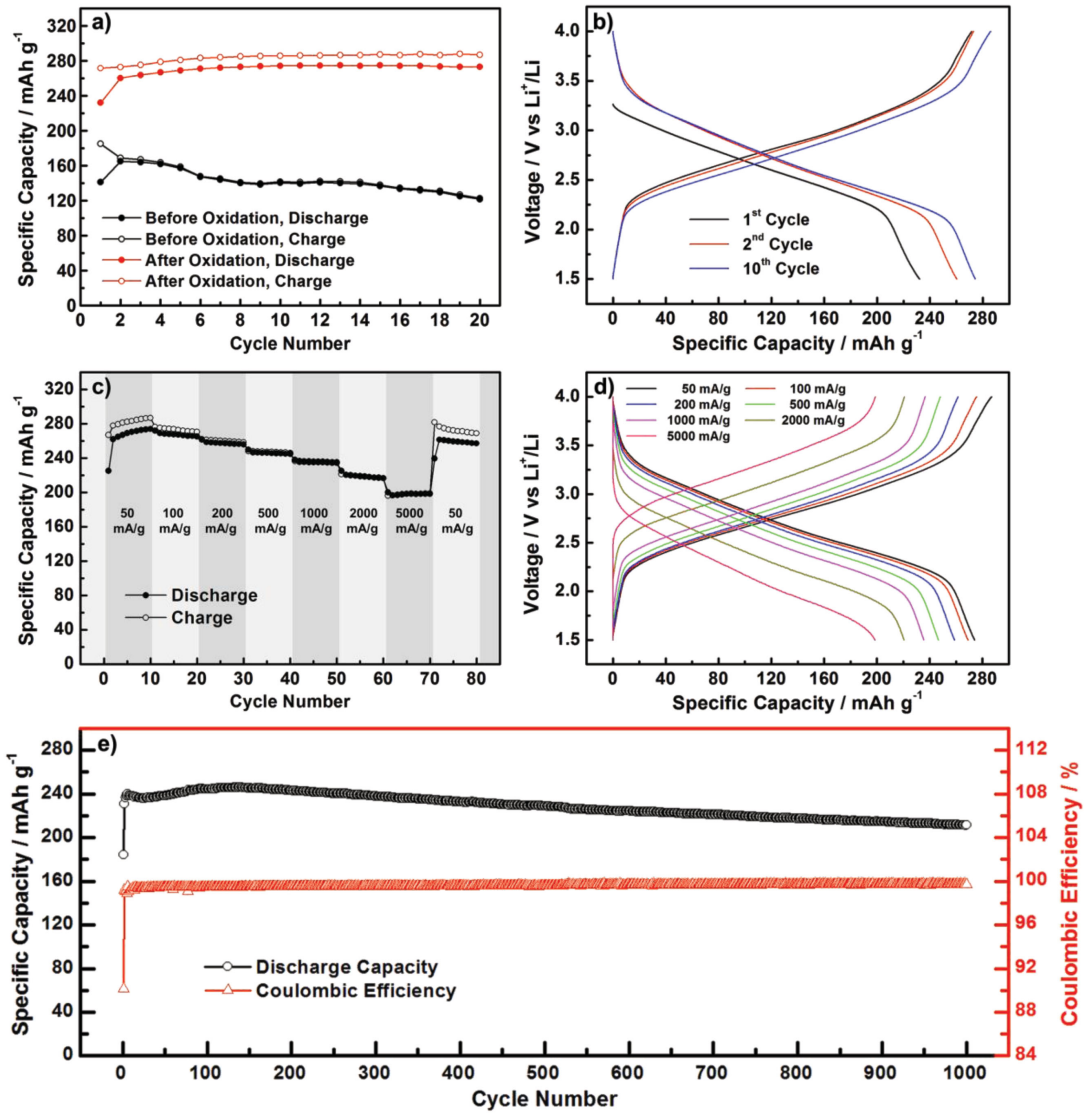
a) Discharge/charge capacity profiles versus cycle number under 50 mA g^–1^ for PBQS samples before and after oxidation. b) Voltage profiles of the 1st, 2nd, and 10th cycle for PBQS after oxidation. c) Discharge/charge capacity profiles versus cycle number under sequentially changed current rate from 50 to 5000 mA g^–1^. d) Corresponding voltage profiles under different current rates. e) Long‐term cycling profiles within 1000 cycles under a current rate of 500 mA g^–1^.

For the comprehensive electrochemical performance of Li–PBQS battery with 1 m LiTFSI/DOL + DME electrolyte, we first concern about the practical reversible capacity of PBQS. Based on the two‐electron reaction of the perfect PBQS structure (Scheme 1b), its theoretical capacity is calculated to be as high as 388 mA h g^–1^. But for our practical PBQS structure, based on the O content (18.3%) coming from both benzoquinone and hydroquinone units [deducting O content in residual NMP (≈0.7%) and absorbed H_2_O (≈3.6%) from the total O content (22.6%) measured by elemental analysis], the theoretical capacity is reduced to 307 mA h g^–1^. At a current rate of 50 mA g^–1^, our PBQS product shows a reversible specific capacity of 275 mA h g^−1^ after oxidation, or only 165 mA h g^−1^ before oxidation (**Figure** **3**a), reaching 90% or 54% of the theoretical value, respectively. We can conclude from this result that benzoquinone units in PBQS can be fully utilized, and the abundance is greatly increased after oxidation. The conversion from hydroquinone to benzoquinone can not only increase the reversible capacity of PBQS, but also improve the cycling stability because hydroquinone groups may release protons to react with the electrolyte and Li anode. Figure 3b shows the discharge/charge curves of the 1st, 2nd, and 10th cycle for PBQS after oxidation. The large capacity gap (40 mA h g^–1^) between the initial discharge process (232 mA h g^–1^) and the next charge process (272 mA h g^–1^), indicates the electrochemical oxidation of remaining hydroquinone groups, thus then a much higher discharge capacity of 260 mA h g^–1^ can be released in the second cycle. Similarly, the discharge capacity is gradually increased in the following cycles to reach a stable value (275 mA h g^–1^) at about the 10th cycle.

Figure 3c,d shows the rate performance of the final PBQS product. Relative to the reversible capacity of 274 at 50 mA g^–1^, the capacity retention is 98%, 94%, 90%, 86%, 80%, or 72%, at a current rate of 100, 200, 500, 1000, 2000 or 5000 mA g^–1^, respectively (Figure 3c). For the corresponding discharge/charged curves shown in Figure 3d, the electrochemical polarization in fast‐discharge/charge process is not serious that the average discharge voltage can still remain 2.20 V even at a high current rate of 5000 mA g^–1^ (thus the power density is 11 kW kg^–1^), releasing a large capacity of 198 mA h g^–1^ in less than 2.5 min. Such excellent high rate performance was rarely seen for either organic or inorganic electrode materials, especially considering that the electrode was fabricated without special techniques such as compositing with carbon nanotube^36^ or graphene,^37^ and the active material loading (60%, 1–2 mg cm^–2^) is not low for insulating organic materials. We ascribe the outstanding rate capability mainly to the fast redox kinetics of quinone groups,^21^, ^37^ which is much superior to the lithium ion diffusion kinetics in the bulk particles of inorganic cathodes. Figure 3e shows the long‐term cycling performance of PBQS within 1000 cycles at a current rate of 500 mA g^–1^. After the initial capacity promotion (from 184 to 231 mA h g^–1^) and the following slow activation process, the reversible capacity reaches the highest value of 246 mA h g^–1^ at about the 140th cycle and then starts to slowly decrease with a decay rate of 0.04 mA h g^–1^ cycle^–1^, and thus the capacity still remains 86% at the 1000th cycle. We believe such cycling stability is much better than that of most reported organic electrode materials and can well satisfy the demand of practical use. It should be noted that, the Coulombic efficiency is only ≈95% at a low current rate of 50 mA g^–1^ (Figure 3a,c), but can be gradually improved to approximately 100% by increasing the current rate (Figure 3c). For example, it is stabilized at above 99.5% after initial several cycles in the long‐term cycling at 500 mA g^–1^ (Figure 3e). We ascribe this abnormal behavior to the side reaction in the cathode during charge process, which is probably the irreversible and continuous oxidation of the electrolyte in the presence of residual O–H groups and/or H_2_O (especially, DOL is not stable at high voltage). This side reaction may possess much slower reaction kinetics than benzoquinone units, so it can be inhibited at large current rate, leading to a high Coulombic efficiency. Based on above data and to our knowledge, PBQS represents the highest level in comprehensive battery performance of organic cathode materials, including not only high energy density, but also high rate capability and high cycling stability.

### 2.4 Na–PBQS Battery

Na‐ion battery is recognized as an ideal alternative to Li‐ion battery in recent years, in concern of the resource abundance of Li and Na element on the earth.^31^ However, it is not as easy as expected to simply transplant the inorganic Li intercalation compounds to Na intercalation electrodes, because of their crystal structure difference caused by the large ionic radius difference between Li^+^ and Na^+^. In this respect, organic electrode materials, especially those based on conjugated‐carbonyl, show their special superiority that Li‐free materials can be directly applied in rechargeable Na batteries,^24^ while Li‐containing materials can also be applied after substituting Li by Na.^25^, ^26^, ^27^, ^28^ To validate our assumption, we have also tested the electrochemical performance of PBQS in 1 m NaTFSI/DOL + DME electrolyte with Na anode.

**Figure 4:**
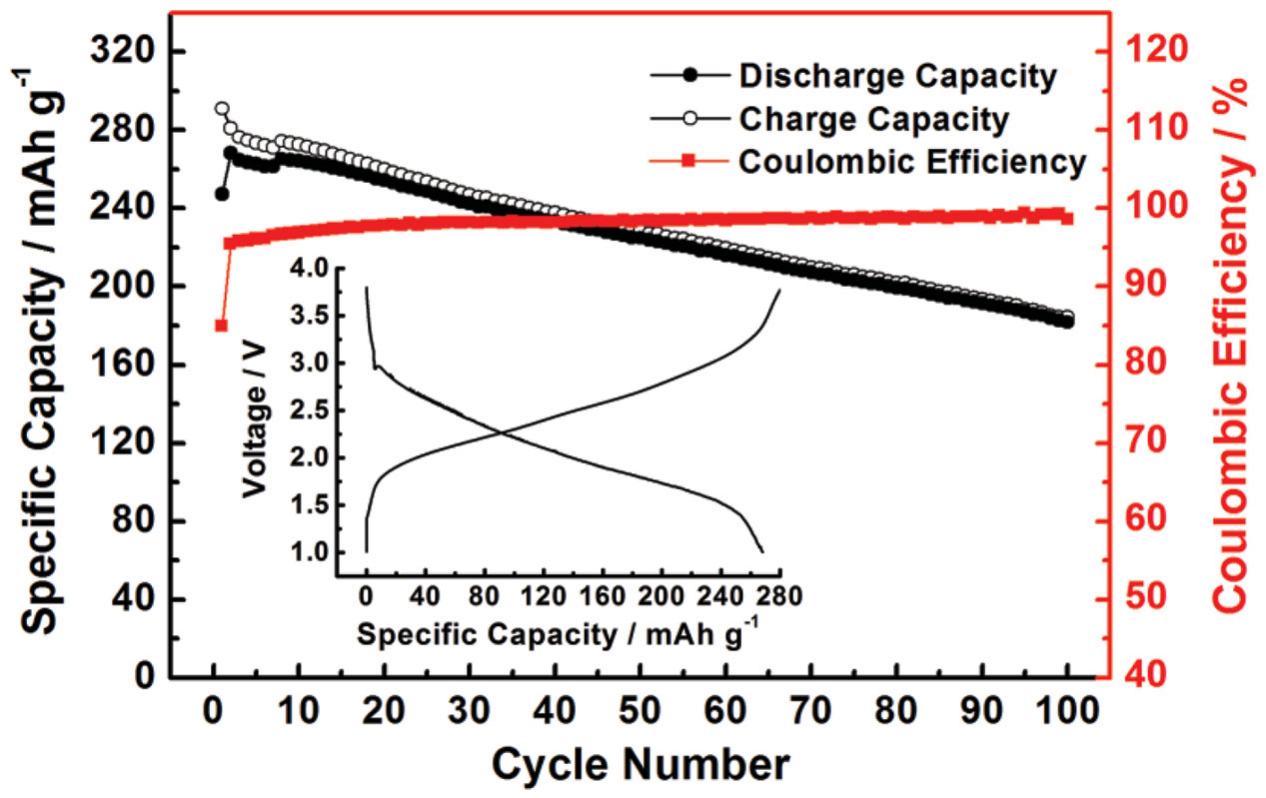
Cycling performance of PBQS within 100 cycles in 1 m NaTFSI/DOL + DME electrolyte, under a current rate of 50 mA g^–1^. Inserted graph shows the typical discharge/charge curves (1.0–3.8 V, 2nd cycle).

**Figure** **4** shows the cycling stability of Na–PBQS battery and the typical discharge/charge curves (2nd cycle). Under the same current rate of 50 mA g^–1^, both the initial and maximum discharge capacity (247 and 268 mA h g^–1^, respectively) are approximate to those of Li–PBQS battery (232 and 275 mA h g^–1^), confirming that benzoquinone units in PBQS can be also fully utilized in spite of much large ionic radius of Na^+^. The discharge/charge curves are also similar to those of Li–PBQS battery, except the lowered average discharge voltage (2.08 V) as expected. Based on above data, PBQS can also achieve a high energy density of 557 W h kg^−1^ (2.08 V × 268 mA h g^−1^) in Na battery, which is superior to that of most inorganic cathodes for Na‐ion batteries.^31^ The cycling stability is not as satisfactory as in Li battery, concluded from the fact that the discharge capacity starts to continuously decrease after the 2nd cycle at a decay rate of 0.88 mA h g^–1^ cycle^–1^ and thus the capacity remains only 68% after 100 cycles. For the rate performance of Na–PBQS battery, it is found the charge capacity becomes larger and instable when the current rate is above 200 mA g^−1^ (Figure S4, Supporting Information), which we ascribe to the inner short circuit caused by sodium dendrites formed in charge process at large current. We suspect there is probably some compatibility problem between Na anode and the 1 m NaTFSI/DOL + DME electrolyte, after all, this electrolyte is barely used in previously reported Na‐ion batteries. Therefore, we attempted to improve the electrochemical performance of Na–PBQS battery by adopting a more commonly used electrolyte for Na‐ion batteries, 1 m NaPF_6_/EC + DEC (Figure S5, Supporting Information). Unfortunately, both the reversible capacity and cycling stability become even poorer, while 1 m LiPF_6_/EC + DEC electrolyte for Li–PBQS battery shows similar result (Figure S5, Supporting Information), which together prove the incompatibility of such kind of electrolyte with PBQS cathode.

### 2.5 Na–PBQS Battery Versus Li–PBQS Battery

**Figure 5:**
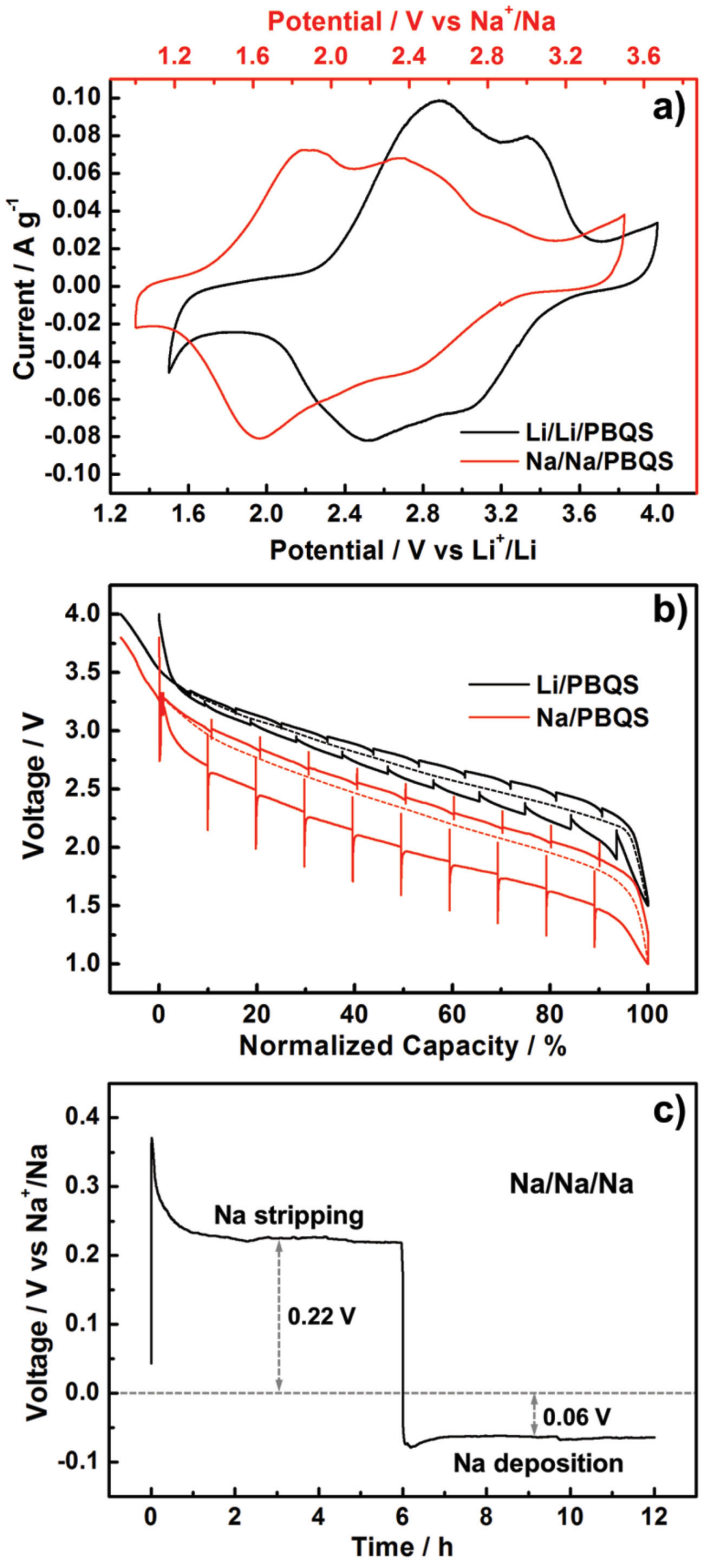
a) Typical CV curves of Li/Li/PBQS and Na/Na/PBQS three‐electrode battery at a scan rate of 0.1 mV s^–1^, in voltage range of 1.5–4.0 V versus Li^+^/Li and 1.0–3.5 V versus Na^+^/Na, respectively. b) Quasi‐open‐circuit voltage (QOCV) profiles of Li/PBQS (1.5–4.0 V) and Na/PBQS (1.0–3.8 V) coin cells at a current rate of 50 mA g^–1^, with a relaxation time of 2 h after each discharge or charge step of 0.5 h. c) Voltage profiles of Na/Na/Na three‐electrode battery at a current density of 50 μA cm^–1^, to show the overpotential of Na electrode. For all the above tests, 1 m LiTFSI/DOL + DME electrolyte was used for Li battery, while 1 m NaTFSI/DOL + DME electrolyte was used for Na battery.

We were curious about the electrochemical behavior difference between Li–PBQS battery and Na–PBQS battery and investigated it by many electrochemical techniques (**Figure** **5**). Figure 5a shows the typical CV curves of Li/Li/PBQS and Na/Na/PBQS three‐electrode battery at a scan rate of 0.1 mV s^–1^. The similarity of CV curves verifies the same redox reaction mechanism, with either Li^+^ or Na^+^ as counterion. The two pairs of redox peaks can be assigned to the well‐known two‐step reaction of quinone unit (Q/Q^•–^ and Q^•–^/Q^2–^), and the serious overlapping is mainly due to peak broadening effect of polymerization, which was observed in many other polymer electrode materials.^12^, ^21^ After eliminating the anode polarization by using a reference electrode and deducting the standard potential gap between Li^+^/Li and Na^+^/Na couple (0.33 V) by normalizing the potential axis, it is found the absolute redox potential of PBQS in Na battery still shifts to the negative side by about 0.3 V, which indicates larger ionic radius of Na^+^ brings about larger energy barrier to overcome in the reduction of PBQS.

Figure 5b shows the quasi‐open‐circuit voltage (QOCV) data of Li–PBQS and Na–PBQS coin cells. At a current rate of 50 mA g^–1^, the electrochemical polarization of Li–PBQS system is very small in either discharge or charge process, benefitting from the high redox reversibility of quinone. However, the gap between average discharge and charge voltage is obviously increased from 0.17 V for Li battery to 0.45 V for Na battery. Besides the large overpotential between the actual discharge curve and the QOCV curve, an obvious voltage hysteresis is also observed that a negative or positive overpotential appears at the very beginning of each discharge or charge step after 2 h relaxation, respectively. We suspected this abnormal phenomenon is related to the stripping/deposition reaction of Na anode in 1 m NaTFSI/DOL + DME electrolyte, so we tested the discharge/charge behavior of Na electrode using a Na/Na/Na three‐electrode battery (Figure 5c). At a current density of 50 μA cm^–1^ (approximate to 50 mA g^–1^ for PBQS cathode), the initial charge and next discharge process of Na electrode (corresponding to the discharge and charge process of PBQS cathode, respectively), show stable overpotentials of 0.22 and 0.06 V after the voltage hysteresis at the beginning. The voltage hysteresis is probably because of the large energy barrier for breaking the thick passivation layer on Na surface,^38^ especially in Na stripping process. The total overpotential of Na anode (0.22 V + 0.06 V = 0.28 V) agrees well with the increase of voltage gap from Li–PBQS battery to Na–PBQS battery (0.45 V – 0.17 V = 0.28 V), which declares that the polarization of Na anode is the dominating origin of the relatively poor reversibility of Na–PBQS battery, and may also responsible for the unsatisfactory cycling stability. Therefore, we believe a more appropriate electrolyte compatible with both Na anode and PBQS cathode may significantly improve the electrochemical performance of Na–PBQS battery, which is still under investigation. From the above discussion, we can draw the conclusion that, if considering only the cathode side, replacing Li^+^ with Na^+^ will only lower the absolute redox potential, but will not obviously affect the utilization and reversibility of PBQS electrode.

## 3 Conclusion

In summary, we developed a novel polymer cathode material by linking benzoquinone units with thioether bonds, namely PBQS, which can essentially solve the dissolution problem while largely maintain the high energy density of benzoquinone monomer. Although the chemical synthesis of benzoquinone polymer is difficult due to its high reactivity, PBQS is successfully synthesized with high yield through a polymerization–oxidation approach using DCBQ and Li_2_S as raw materials. As a polymer cathode for rechargeable Li battery, PBQS shows different discharge/charge behavior from BQ monomer, which is well illustrated by an energy level diagram obtained by DFT calculation. Benefiting from the high redox potential, high theoretical capacity and fast reaction kinetics of benzoquinone units, as well as intrinsic insolubility of polymer chains linked by thioether bonds, PBQS demonstrates superior comprehensive battery performance, including not only high energy density (2.67 V × 275 mA h g^−1^ = 734 W h kg^−1^) exceeding those of commercial inorganic cathodes (e.g., LiCoO_2_ and LiFePO_4_) and other insoluble organic cathodes, but also stable long‐term cycling performance (1000 cycles, 86%), as well as excellent fast‐discharge/charge ability (5000 mA g^–1^, 72%). Due to the specialty of conjugated‐carbonyl, PBQS was also directly applied as cathode for rechargeable Na battery, showing similar discharge/charge behavior and gaining high energy density (2.08 V × 268 mA h g^−1^ = 557 W h kg^−1^) as well. The unsatisfactory cycling and rate performance of Na–PBQS battery are mainly ascribed to the poor compatibility between Na anode and the 1 m NaTFSI/DOL + DME electrolyte. Since the structure of our PBQS product is not perfect (containing residual hydroquinone units, water and NMP), there is still room to further improve the energy density to nearly 1000 W h kg^−1^ (in Li battery) by optimizing the synthesis conditions. We also look forward to better Na–PBQS battery performance by optimizing the electrolytes. We believe, our preliminary investigations on PBQS show the enormous possibilities of organic cathode materials, encouraging researchers to explore more and more high‐energy organic cathodes, toward the next generation of high‐performance, low‐cost, and sustainable energy storage devices.

## Supporting information

As a service to our authors and readers, this journal provides supporting information supplied by the authors. Such materials are peer reviewed and may be re‐organized for online delivery, but are not copy‐edited or typeset. Technical support issues arising from supporting information (other than missing files) should be addressed to the authors.

Supplementary

## References

[1] M. Armand , J.‐M. Tarascon , Nature 2008, 451, 652. 18256660 10.1038/451652a

[2] R. Van Noorden , Nature 2014, 507, 26. 24598624 10.1038/507026a

[3] M. S. Whittingham , Chem. Rev. 2014, 114, 11414. 25354149 10.1021/cr5003003

[4] P. G. Bruce , S. A. Freunberger , L. J. Hardwick , J.‐M. Tarascon , Nat. Mater. 2012, 11, 19. 10.1038/nmat3191 22169914

[5] A. Manthiram , Y. Fu , S.‐H. Chung , C. Zu , Y.‐S. Su , Chem. Rev. 2014, 114, 11751. 25026475 10.1021/cr500062v

[6] A. C. Luntz , B. D. McCloskey , Chem. Rev. 2014, 114, 11721. 25376975 10.1021/cr500054y

[7] P. Poizot , F. Dolhem , Energy Environ. Sci. 2011, 4, 2003.

[8] Y. Liang , Z. Tao , J. Chen , Adv. Energy Mater. 2012, 2, 742.

[9] Z. Song , H. Zhou , Energy Environ. Sci. 2013, 6, 2280.

[10] H. Chen , M. Armand , G. Demailly , F. Dolhem , P. Poizot , J.‐M. Tarascon , ChemSusChem 2008, 1, 348. 18605101 10.1002/cssc.200700161

[11] H. Chen , M. Armand , M. Courty , M. Jiang , C. P. Grey , F. Dolhem , J.‐M. Tarascon , P. Poizot , J. Am. Chem. Soc. 2009, 131, 8984. 19476355 10.1021/ja9024897

[12] Z. Song , H. Zhan , Y. Zhou , Chem. Commun. 2009, 448. 10.1039/b814515f 19137181

[13] M. Yao , H. Senoh , S.‐i. Yamazaki , Z. Siroma , T. Sakai , K. Yasuda , J. Power Sources 2010, 195, 8336.

[14] W. Choi , D. Harada , K. Oyaizu , H. Nishide , J. Am. Chem. Soc. 2011, 133, 19839. 22011047 10.1021/ja206961t

[15] H. Senoh , M. Yao , H. Sakaebe , K. Yasuda , Z. Siroma , Electrochim. Acta 2011, 56, 10145.

[16] M. Yao , S.‐i. Yamazaki , H. Senoh , T. Sakai , T. Kiyobayashi , Mater. Sci. Eng. B 2012, 177, 483.

[17] Y. Hanyu , Y. Ganbe , I. Honma , J. Power Sources 2013, 221, 186.

[18] Y. Liang , P. Zhang , S. Yang , Z. Tao , J. Chen , Adv. Energy Mater. 2013, 3, 600.

[19] W. Huang , Z. Zhu , L. Wang , S. Wang , H. Li , Z. Tao , J. Shi , L. Guan , J. Chen , Angew. Chem. Int. Ed. 2013, 52, 9162. 10.1002/anie.201302586 23825051

[20] Z. Zhu , M. Hong , D. Guo , J. Shi , Z. Tao , J. Chen , J. Am. Chem. Soc. 2014, 136, 16461. 25383544 10.1021/ja507852t

[21] Z. Song , Y. Qian , X. Liu , T. Zhang , Y. Zhu , H. Yu , M. Otani , H. Zhou , Energy Environ. Sci. 2014, 7, 4077.

[22] X. Han , C. Chang , L. Yuan , T. Sun , J. Sun , Adv. Mater. 2007, 19, 1616.

[23] Z. Song , H. Zhan , Y. Zhou , Angew. Chem. Int. Ed. 2010, 49, 8444. 10.1002/anie.201002439 20862664

[24] H.‐g. Wang , S. Yuan , D.‐l. Ma , X.‐l. Huang , F.‐l. Meng , X.‐b. Zhang , Adv. Energy Mater. 2014, 4, 1301651.

[25] M. Armand , S. Grugeon , H. Vezin , S. Laruelle , P. Ribière , P. Poizot , J.‐M. Tarascon , Nat. Mater. 2009, 8, 120. 19151701 10.1038/nmat2372

[26] L. Zhao , J. Zhao , Y.‐S. Hu , H. Li , Z. Zhou , M. Armand , L. Chen , Adv. Energy Mater. 2012, 2, 962.

[27] S. Wang , L. Wang , K. Zhang , Z. Zhu , Z. Tao , J. Chen , Nano Lett. 2013, 13, 4404. 23978244 10.1021/nl402239p

[28] S. Wang , L. Wang , Z. Zhu , Z. Hu , Q. Zhao , J. Chen , Angew. Chem. Int. Ed. 2014, 53, 5892. 10.1002/anie.201400032 24677513

[29] T. Nokami , T. Matsuo , Y. Inatomi , N. Hojo , T. Tsukagoshi , H. Yoshizawa , A. Shimizu , H. Kuramoto , K. Komae , H. Tsuyama , J.‐i. Yoshida , J. Am. Chem. Soc. 2012, 134, 19694. 23130634 10.1021/ja306663g

[30] Y. Liang , P. Zhang , J. Chen , Chem. Sci. 2013, 4, 1330.

[31] N. Yabuuchi , K. Kubota , M. Dahbi , S. Komaba , Chem. Rev. 2014, 114, 11636. 25390643 10.1021/cr500192f

[32] H.‐D. Becker , J. Org. Chem. 1965, 30, 982.

[33] P. Piaggio , C. Cuniberti , G. Dellepiane , E. Campani , G. Gorini , G. Masetti , M. Novi , G. Petrillo , Spectrochim. Acta, Part A 1989, 45, 347.

[34] K. Liu , J. Zheng , G. Zhong , Y. Yang , J. Mater. Chem. 2011, 21, 4125.

[35] I. B. Bersuker , Chem. Rev. 2013, 113, 1351. 23301718 10.1021/cr300279n

[36] M. Lee , J. Hong , H. Kim , H.‐D. Lim , S. B. Cho , K. Kang , C. B. Park , Adv. Mater. 2014, 26, 2558. 24488928 10.1002/adma.201305005

[37] Z. Song , T. Xu , M. L. Gordin , Y.‐B. Jiang , I.‐T. Bae , Q. Xiao , H. Zhan , J. Liu , D. Wang , Nano Lett. 2012, 12, 2205. 22449138 10.1021/nl2039666

[38] S.‐Y. Ha , Y.‐W. Lee , S. W. Woo , B. Koo , J.‐S. Kim , J. Cho , K. T. Lee , N.‐S. Choi , ACS Appl. Mater. Interfaces 2014, 6, 4063. 24559269 10.1021/am405619v

