# Supplementary material for: Poly(benzoquinonyl sulfide) as a High‐Energy Organic Cathode for Rechargeable Li and Na Batteries
Source: Adv Sci (Weinh). 2015 Jun 8;2(9):1500124. doi: 10.1002/advs.201500124 (PMC5115381; doi:10.1002/advs.201500124)
Supplement: Supplementary file 1 — Supplementary [file ADVS-2-0n-s001.pdf]

Copyright WILEY-VCH Verlag GmbH & Co. KGaA, 69469 Weinheim, Germany, 2015.

ADVANCED SCIENCE
Open Access

Supporting Information

for *Adv. Sci.*, DOI: 10.1002/advs.201500124

Poly(benzoquinonyl sulfide) as a High-Energy Organic Cathode for Rechargeable Li and Na Batteries

*Zhiping Song, Yumin Qian, Tao Zhang, Minoru Otani, and Haoshen Zhou**

Copyright WILEY-VCH Verlag GmbH & Co. KGaA, 69469 Weinheim, Germany, 2015.

Supporting Information

Poly(benzoquinonyl sulfide) as a High-Energy Organic Cathode for Rechargeable Li and Na Batteries

*Zhiping Song, Yumin Qian, Tao Zhang, Minoru Otani and Haoshen Zhou**

EXPERIMENTAL DETAILS

Synthesis Procedures. In a typical synthesis of poly(benzoquinonyl sulfide) (PBQS), 2,5-dichloro-1,4-benzoquinone (DCBQ, 1.77 g, 0.010 mol) was first dissolved in 30 mL 1-methyl-2-pyrrolidone (NMP). Anhydrous Li_2S (0.69 g, 0.015 mol) was then added into the solution. The mixture was heated at 160 °C for 12 hours under stirring in nitrogen atmosphere. After cooling to room temperature, the viscous mixture was poured into 100 mL 0.1 M HCl solution to precipitate the product. The precipitate was filtrated and washed with water until the filtrate is colorless. For purification, the filter cake was completely dissolved in 100 mL 0.1 M LiOH solution. Concentrated HCl was added into the solution drop by drop until the pH value turned to approx. 1, generating a large amount of precipitate. The mixture was centrifuged and washed with 0.1 M HCl solution for twice, and then ethanol containing 0.1 M HCl for several times until the solution is nearly colorless. The product was dried at 80 °C in vacuum, obtaining a black solid with high yield of approx. 90%.

As-synthesized PBQS was further treated to oxidize hydroquinone groups to quinone groups. In a typical process, 0.80 g of above product was homogeneously dispersed in 40 mL tetrahydrofuran (THF) through probe sonication. Excess 2,3-dichloro-5,6-dicyano-1,4-benzoquinone (DDQ, 1.60 g) was then added into the dispersion. After stirring at room temperature for 2 days, the mixture was centrifuged and washed with THF for several times

until the solution is nearly colorless. The product was dried at 80 °C in vacuum, obtaining a black solid with yield close to 100%.

Material Characterizations. Elemental analysis (EA) of CHN, O and Cl were conducted on EA1110 element analyzer (CE Instruments, Italy), EA1112 element analyzer (CE Instruments, Italy) and ICS-2000 ion chromatography (Dionex, Japan), respectively. EA of S element was conducted on a combustion ion chromatography. FTIR spectroscopy was recorded using KBr pellet on a FT/IR-6200 spectrometer (JASCO, Japan) in wavenumber range of 400–4000 cm^{-1}. TG (thermogravimetric) analysis was performed on a 2020S TG-DTA Analyzer (MAC Science, Japan) in temperature range of 20–600 °C at a heating rate of 5 °C min^{-1}. XPS (X-ray photoelectron spectroscopy) analysis was performed on a VG Theta Probe spectrometer (Thermo Fisher Scientific, USA) with Al Kα X-ray source.

Electrochemical Tests. CR2032-type coin cells used for battery tests consisted of lithium or sodium foil anode, Celgard 2400 membrane, glass fiber separator, and the cathode film. The cathode film was prepared by homogeneously mixing the active material, conductive carbon (Ketjenblack EC-600JD) and polytetrafluoroethylene (PTFE) binder, with weight ratio of 6: 3: 1 for PBQS or 3: 6: 1 for BQ, before being pressed onto aluminum mesh (100 mesh) as current collector. The active material loading on electrode (ϕ=8 mm) was typically 1–2 mg cm^{-2}. Unless otherwise specified, the electrolyte was 1 M $LiN(CF_3SO_2)_2$ (LiTFSI) or $NaN(CF_3SO_2)_2$ (NaTFSI) solution in a mixed solvent of 1,3-dioxolane (DOL) and dimethoxyethane (DME) (1:1, v/v), for Li battery or Na battery, respectively. Otherwise, 1 M $LiPF_6$ or $NaPF_6$ solution in a mixed solvent of ethylene carbonate (EC) and diethyl carbonate (DEC) (1:1, v/v) was used for comparison. The galvanostatic discharge/charge tests were performed on a HJ-SD8 battery test system (Hokuto Denko, Japan) using coin cells or three-electrode cells (Na/Na/Na, representing reference/counter/working electrode, similarly

hereinafter) under different conditions. CV (cyclic voltammetry) measurements were conducted on a Solartron 1287/1260 electrochemical workstation using three-electrode cells (Li/Li/PBQS or Na/Na/PBQS) at a scan rate of 0.1 mV s^{-1}. Quasi-open-circuit voltage (QOCV) measurements were also conducted on above instrument using coin cells at a current rate of 50 mA g^{-1}, with a relaxation time of 2 hours after each discharge or charge step of 0.5 hour. All the electrochemical tests were performed at room temperature.

DFT Calculations. Electron configurations of all the molecules were calculated by DFT method within the framework of the Gaussian 09 package. The standard Pople basis set, 6-31G(d,p), combined with the Lee–Yang–Parr exchange correlation functional (B3LYP) was used for all calculations. For each molecule, the geometry was fully optimized to achieve the lowest total energy before energy level calculation, and all possible spin multiplicities were explored (S=0, 1, 2), among which we chose the one with the lowest energy for comparison between different molecules.

Table S1. Characteristic IR band assignments of PBQS samples before oxidation and after oxidation. The suffix follow the wavenumber (cm^{-1}) means the strength and shape of the peak: s, strong; m, medium; w, weak; br, broad.

Vibration Type	Before Oxidation	After Oxidation
O—H of absorbed H_2O, stretching	3427s,br	3434s,br
O—H of hydroquinone, stretching	3129s,br	
conjugated C=O and C=C, stretching	1629s	1647s
sulfur substituted benzene ring, stretching	1523w, 1495w, 1452s, 1401s	1532m,1495m, 1454m,1401m
C—OH, stretching	1239s	1216s
Ar—S, stretching	1127w	1128w

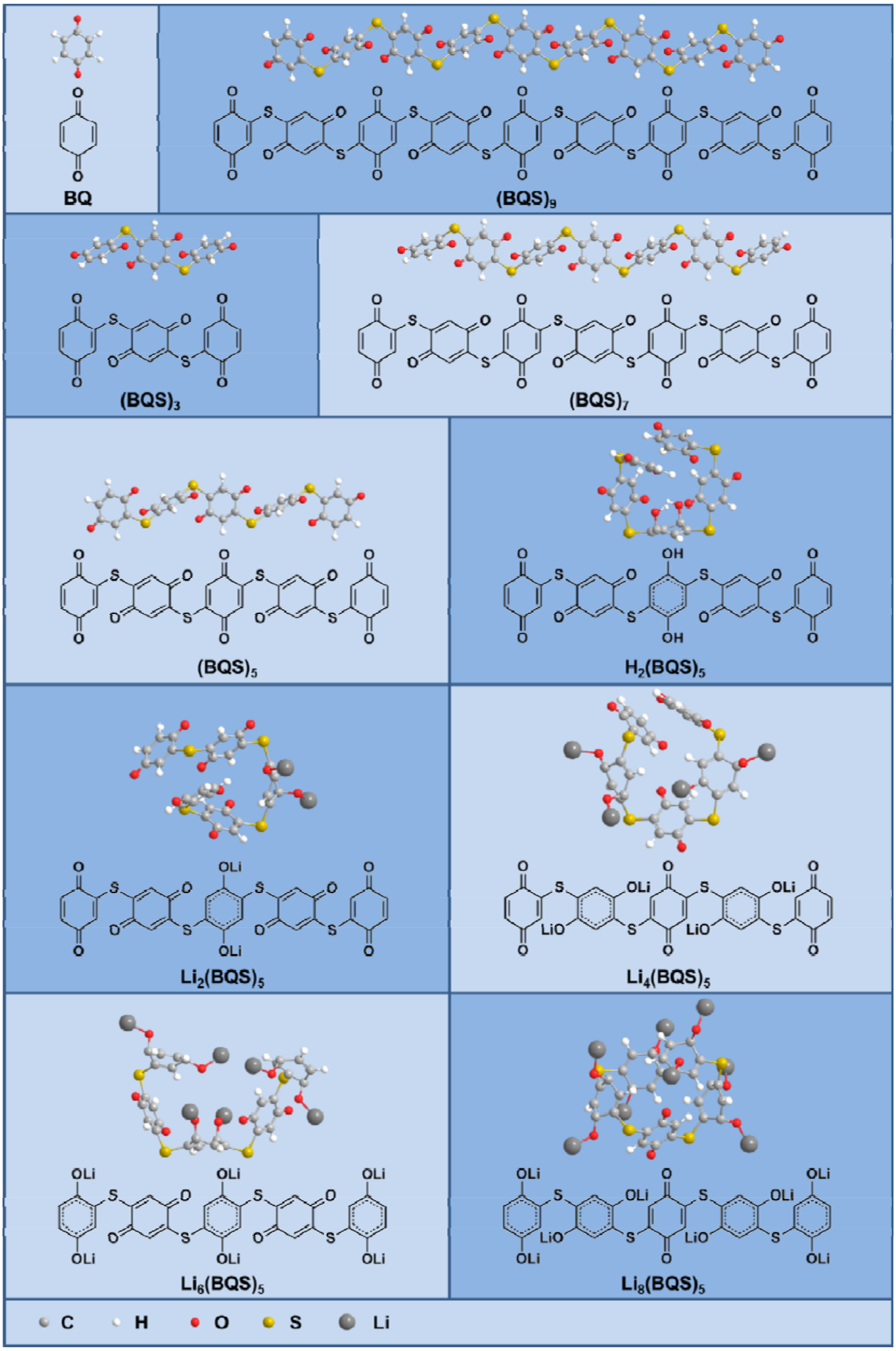

Figure S1. Structural formulas and optimized geometries of related molecules for DFT calculations.

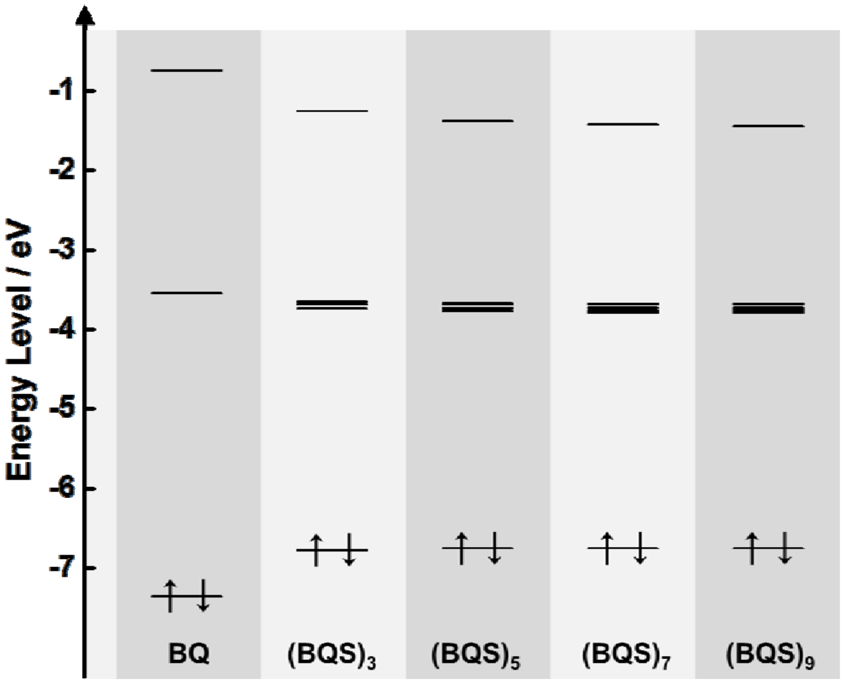

Figure S2. The calculated electron configurations of BQ monomer and simulated PBQS in different polymerization degrees [$(BQS)_3$, $(BQS)_5$, $(BQS)_7$, $(BQS)_9$]. All the polymerized molecules show lowered and nearly degenerate orbitals with slightly descending LUMO energies [–3.54, –3.74, –3.77, –3.79 and –3.80 eV for BQ, $(BQS)_3$, $(BQS)_5$, $(BQS)_7$ and $(BQS)_9$, respectively].

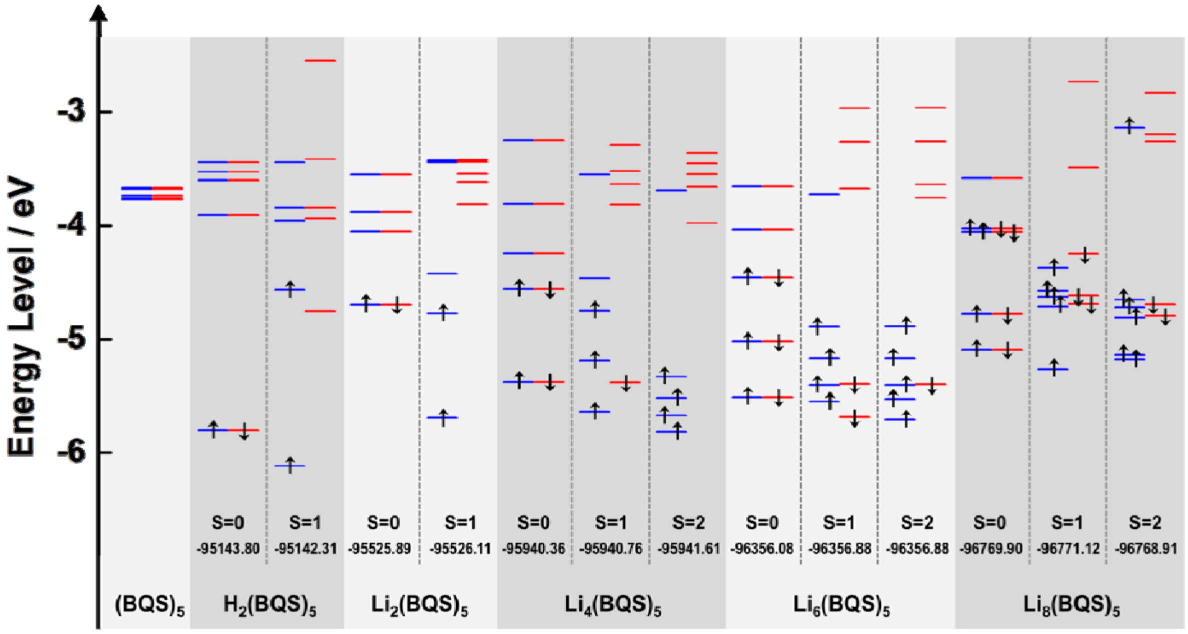

Figure S3. The calculated electron configurations of simulated polymer, $(BQS)_5$, and those with residual hydroquinone groups $[H_2(BQS)_5]$, and in different reduction states $[Li_2(BQS)_5$, $Li_4(BQS)_5$, $Li_6(BQS)_5$ and $Li_8(BQS)_5]$. For each molecule all the possible spin multiplicities are explored (S=0, 1, 2). The values below S are the total energies (eV) of the molecule with corresponding spin states, among which we choose the one with the lowest energy for comparison in Fig. 2b [S=0, S=1, S=2, S=1 and S=1 for $H_2(BQS)_5$, $Li_2(BQS)_5$, $Li_4(BQS)_5$, $Li_6(BQS)_5$ and $Li_8(BQS)_5$, respectively].

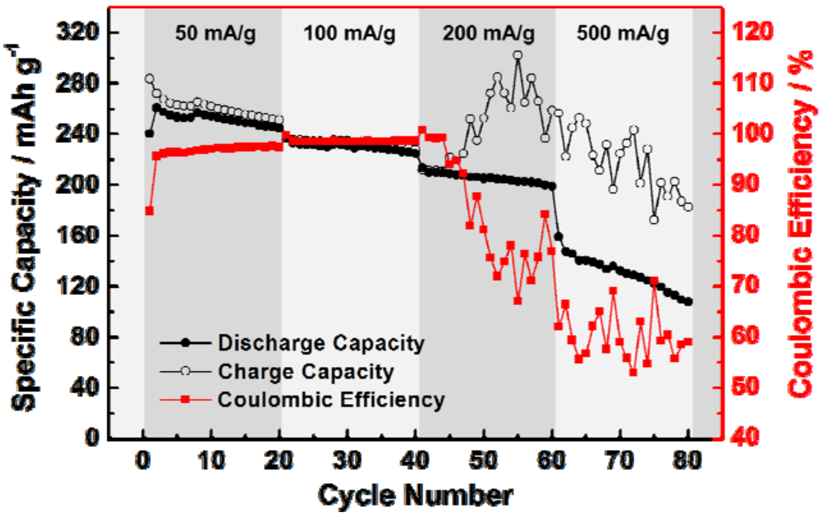

Figure S4. Cycling performance of Na–PBQS battery under sequentially increased current rate from 50 to 500 mA g^{-1}. When the current rate is above 200 mA g^{-1}, the Coulombic efficiency is reduced and instable, which can be ascribed to the inner short circuit caused by sodium dendrites formed in charge process at large current.

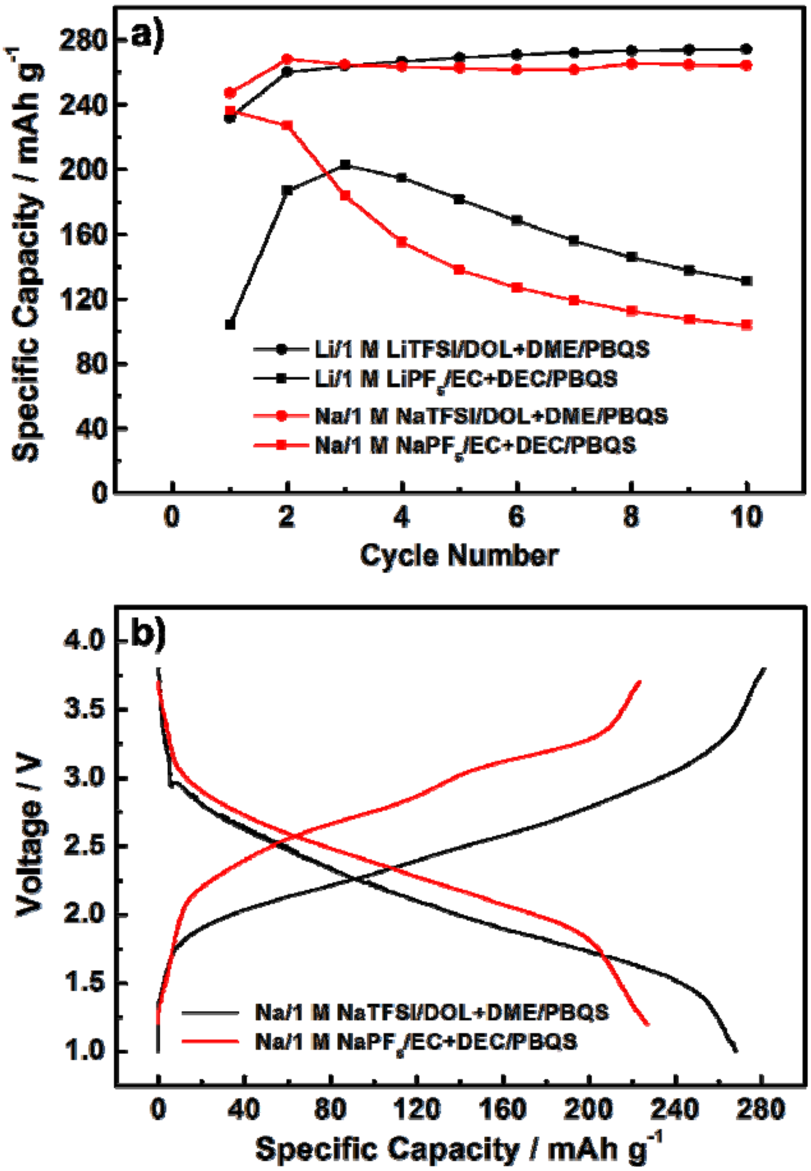

Figure S5. a) Discharge capacity profiles vs. cycle number of Li–PBQS or Na–PBQS battery with different electrolyte, under a current rate of 50 mA g^{-1}. b) Typical voltage profiles (2nd cycle) of Na–PBQS batteries with different electrolytes.

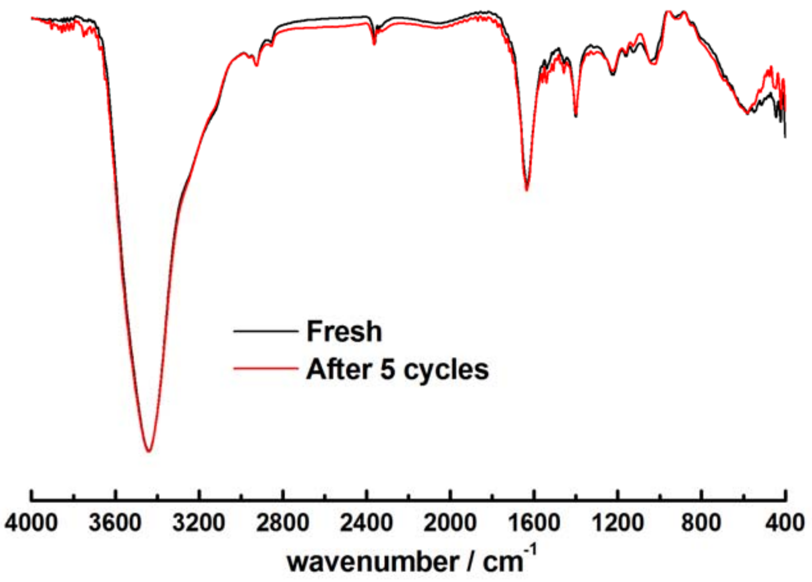

Figure S6. FTIR spectra of fresh PBQS electrode (PBQS: KB: PTFE = 6: 3: 1) and that after five discharge/charge cycles (both are in charged state). The spectra are nearly the same, indicating that the structure of PBQS is rather stable during discharge/charge process.
